# Maternal exposure to intimate partner violence and uptake of maternal healthcare services in Ethiopia: Evidence from a national survey

**DOI:** 10.1371/journal.pone.0273146

**Published:** 2022-08-18

**Authors:** Seman K. Ousman, Mekdes K. Gebremariam, Johanne Sundby, Jeanette H. Magnus

**Affiliations:** 1 St Paul’s Hospital Millennium Medical College (SPHMMC), School of Public Health, Addis Ababa, Ethiopia; 2 Institute of Health and Society, HELSAM, Department of Community Medicine and Global Health, Faculty of Medicine, University of Oslo, Oslo, Norway; 3 Center for Global Health, Faculty of Medicine, University of Oslo, Oslo, Norway; 4 Department of Global Community Health and Behavioral Sciences, Tulane School of Public Health and Tropical Medicine, New Orleans, Louisiana, United States of America; University of Salamanca, SPAIN

## Abstract

**Background:**

Women exposed to Intimate Partner Violence (IPV) often do not utilize maternal health care optimally both because of stigma and other social problems. The current study aims to explore an association between maternal healthcare seeking and violence exposure among Ethiopian women and to assess if educational attainment and wealth status moderate this association.

**Methods:**

The analyses included 2836 (weighted) currently married women with one live birth. We focus on the five years preceding the 2016 Ethiopian Demographic and Health Survey (EDHS) who participate, in the domestic violence sub-study. Exposure was determined by maternal reports of physical, emotional, sexual IPV or any form of IPV. The utilization of antenatal care (ANC) and place of delivery were used as proxy outcome variables for uptake of skilled maternal healthcare utilization. Women’s education attainment and wealth status were selected as potential moderators, as they can enable women with psychological and financial resources to counteract impact of IPV. Multilevel logistic regression analyses were used to explore the association between spousal IPV and maternal health outcomes. Moderation effects by education and wealth status were tested, and the data stratified. Using statistical software Stata MP 16.1, the restricted maximum likelihood method, we obtained the model estimates.

**Results:**

About 27.5% of the women who reported exposure to any form of IPV had a health facility delivery. While 23.4% and 22.4% visited four or more antenatal care services among mothers exposed to emotional IPV and sexual IPV, respectively. After adjusting for potential confounding factors, only the association between maternal exposure to emotional IPV and adequate use of ANC was statistically significant (OR = 0.73, (95% CI:0.56–0.95)). But we found no significant association between IPV and utilization of health facility delivery. Some moderation effects of education and wealth in the association between IPV and maternal healthcare service utilization outcome were found.

**Conclusion:**

Exposure to emotional IPV was associated with poor uptake of maternal health care service utilization for married Ethiopian women. While developing interventions to improve women’s maternal healthcare service use, it is crucial to consider the effects of socio-economic variables that moderate the association especially with the intersection of IPV.

## 1. Background

Intimate Partner Violence (IPV) is defined as: the intentional use of physical force, emotional or sexual abuse, by an intimate partner. IPV is linked to a wide range of health problems that directly or indirectly affects maternal morbidity and mortality [1]. As 30% of women report emotional, physical and/or sexual violence by an intimate partner in their life time, it is a significant global public health problem [2]. Emotionally, physically or sexually abused women report higher rates of health problems including preterm birth, low birth weight, abortion, depression, and HIV/STI infection, [1]. The negative impact of IPV are not limited to women’s health but children in these families are also vulnerable to the physical and mental health consequences of such adverse experiences [3]. Exposure to IPV has an inconsistent and often nonlinear relationship with the uptake of maternal healthcare services. Some studies indicate a negative association of different forms of IPV and the uptake of adequate antenatal care (ANC), skilled delivery care, and postnatal care [4–10], while studies in developing countries conclude that different forms of IPV do not have any significant association with the uptake of skilled maternal healthcare [11, 12].

IPV is common in urban and rural Ethiopian families. One-third (34%) of ever-married women aged 15–49 years report experience of physical, sexual, or emotional IPV [13]. Furthermore, close to one in five women reported at least three forms of marital controlling behaviour from their partner [13]. Living in rural areas, low maternal education and poor household wealth, are strong predictors of IPV [14, 15]. Literature regarding the effects of IPV on women’s health in Ethiopia found associations between IPV and adverse birth outcomes [16], unintended pregnancy [17], pregnancy loss [18], unmet need for family planning [19], depression [20, 21], Human Immuno-deficiency Virus (HIV) status [22], and antiretroviral therapy adherences [23]. Mediation effect of contraceptive use and women’s autonomy on the relationship between IPV and unintended pregnancy has also been documented [24].

In addition, some regional studies’ reported linkages between exposure to IPV and maternal health outcomes in Ethiopia [25–27]. A number of socio-demographic factors are known to influence exposure to IPV, or the uptake of appropriate maternal healthcare services [4, 10, 12, 28]. Other studies have demonstrated that mother’s educational attainment and household wealth status together accounted for a large share of impact on IPV and in the uptake of maternal health services in Ethiopia [29]. Mothers with low educational attainment and low income might have a greater susceptibility to the adverse impacts of IPV than mothers with higher educational attainment and income, who may be protected by greater psychological, social and financial resources. However, no earlier study has explored if there are any moderators of the relationship between different forms of IPV and maternal health outcomes in Ethiopia. The current study aims to understand the effect of IPV on women’s uptake of maternal healthcare services (adequate ANC visits and use of health facility delivery) and whether women’s educational attainment or wealth status moderate any relationship between IPV and maternal health outcome using a large Ethiopian national sample. Determining if education level and wealth status are moderators of the association between IPV and maternal healthcare service would help clarify which women tend to be negatively affected by IPV and assist in tailoring and targeting the intervention to those most likely to respond to specific intervention efforts.

## 2. Materials and methods

### 2.1 Data source

The publically available data of the fourth nationally representative survey of the 2016 Ethiopia Demographic and Health Survey (EDHS) was collected between January–June 2016. The full details of the data collection methods and procedures as well as the standards for protecting the privacy of study participants have been published [13]. IPV information for ever-married women age 15–49 ever reporting exposure of spousal emotional, physical, or sexual violence was collected using a modified and abbreviated version of the Revised Conflict Tactics Scales (CTS2) [30]. After excluding missing values, a total of 3061 (unweighted) ever-married women in reproductive age were considered in this study [13]. Special domestic violence weights were used to make the survey data on violence nationally representative accounting for non-response [31]. The final study sample was further limited to those who were currently married and had at least one live birth in the five years preceding the survey (weighted, n = 2836).

### 2.2. Measurement of variables

#### 2.2.1. Outcome measurement

The analyses in the current study address two maternal healthcare binary outcomes: (1) adequate antenatal care (ANC) use; categorized into four or more visits (≥4) and less than four visits (<4, this included women with no visit), in accordance with the 2002 WHO ANC model [32], which was recommended by the Ethiopian Federal Ministry of Health (FMoH), at the time of initiating this study (This is not the current recommended ANC protocol by WHO which is based on WHO’s 2016 ANC Model prescribing a minimum of eight contacts.) and (2) place of delivery, categorized as home birth or birth at a health facility.

#### 2.2.2. Predictors

The predictor variables were reported as exposure to emotional, physical, sexual IPV or any type of IPV. In the current study, emotional IPV is a composite binary variable based on responses to three questions: Had the husband ever: (1) said or did something to humiliate her in front of others; (2) threatened to hurt or harm her or someone she cared about; or (3) insulted or made her feel bad about herself, with yes (experiencing at least one of these); and (not experiencing any), [13, 28, 33]. Similarly, physical IPV is a composite binary variable based on women’s responses to the questions about whether the husband ever had done any of the seven following acts: (1) push, shake, or throw something; (2) slap; (3) twist arm or pull hair; (4) punch with fist or with something that could be harmful; (5) kick, drag, or beat her up; (6) tried to choke or burn her; (7) threaten or attacked with any material to deliberately hurt her at one point in lives [13, 28, 33]. Sexual IPV was, responding yes to any of these three questions: (1) physically forced to have sex; (2) forced to other sexual acts; (3) forced by threats when she did not want to [13, 28, 33]. Lastly, any IPV, was a composite dichotomous summary measure created from 13 questions (emotional IPV: 3, physical IPV: 7, and sexual IPV:3) to capture the women’s ever experience of any IPV (emotional, physical and/or sexual), grouped as: Yes (‘yes’ responses to any of these 13 questions), and No (‘no’ responses to all of the 13 questions).

#### 2.2.3. Moderators

Based on the literature, two variables–women’s education level and household wealth status were considered as potential moderators [27, 28]. Education level of the woman was based on: the highest level of education attained by the respondent and grouped into two: as None, or Primary and above. Household wealth index; a composite index of household possessions, assets, and amenities, derived using principal component analysis (PCA), and ranked as poorer; poor; middle; rich; and richest. For our analysis, we re-categorized wealth into three categories (poor, middle, rich) [34, 35].

#### 2.2.4. Confounders

Based on the current literature, we included several potential confounding variables. The woman’s self-reported age at the time of the survey, was categorized as younger (15–24 years); middle (25–34 years) and older (35–49 years) as age affects health seeking behaviors, [36]; the order of the last birth closes to the time of the survey; education level of the partner reported as none, primary and above; exposure to mass media (composite variable based on the access to and frequency of use of radio and/or television at least once a week), [37]; decision-making autonomy in making three household decisions (access to health care; large household purchases; and freedom to visit families and relatives), grouped into, low autonomy (no participation in any decision making), medium autonomy (participation in 1 or 2) and high autonomy (participation in all decision making); attitude towards wife beating was created using scenarios: (1) she burns the food; (2) she argues with him; (3) she goes out without telling him; (4) she neglects the children and (5) she refuses to have sex with him. A woman was regarded as accepting violence if she said it was justified for any of these five reasons and as rejecting if she reported that beating was not justified for any reasons [38], and the place of residence at the time of the survey categorized as urban or rural. The regions were defined according to the FMoH as agrarian (Tigray, Amhara, Oromia and SNNPR), pastoralist (Somali, Afar, Gambella, and Benishangul Gumuz regions) and city dwellers (Addis Ababa, Dire Dawa, and Harar).

### 2.3. Statistical analysis

We used bivariate analyses to describe the characteristics of the women in relation to the outcome of interest and each type of IPV along with the Pearson Chi-square (X^2^) test of independence to examine whether there were any significant differences in the sociodemographic characteristics, and the associated *p*-value calculated. Sampling weights were applied for the data when we computed both the bivariate and multivariate analysis to manage the unequal probability of selection between the strata defined by geographical location and for non-responses. We fitted separate random-effects multilevel logistic regression models, for each outcome of interest (ANC and delivery care) using only the variables that were significantly associated with each outcome and type of IPV in the bivariate models.

#### 2.3.1. Modeling binary responses

We used a binary logistic multilevel regression model, as the data was clustered at the survey level (2836 women nested in 626 clusters). Univariate logistic regression was performed to estimate the crude odds ratios (COR), (See, S1 Table). And the 95% confidence intervals (95%CI) of facility delivery or not, and if she had at least four ANC visits or not. Potential predictors and confounders significantly associated with the outcome variables in the univariate analysis were entered in the multilevel logistic regression analysis. We conducted four separate fully adjusted models for each type of IPV (emotional, physical, sexual, and any type of violence) for each outcome variable while controlling for confounders to identify the association between spousal IPV on the use of maternal healthcare services.

To assess any moderating effect of education and/or wealth in the association between exposure to spousal IPV and maternal healthcare services, interactions were checked (interaction between IPV and education/wealth). Finally, analyses of the association between exposure to spousal IPV and maternal healthcare services were stratified by level of education and household wealth status. Prior to the multivariate regression analysis, multi-collinearity was checked using variance inflation factors (VIF) which indicates that there was no multi-collinearity since all variables have VIF <2 and all were considered for the subsequent analysis. In addition, we computed an estimate of intra-cluster correlation coefficient (ICC), which described the amount of variability in the response variables attributable to differences between the clusters. We examined the model fit measured using the Akaike information criteria (AIC). A lower AIC value represents a better model fit [39]. All the statistical analyses were performed using complex sample analysis procedure to allow for adjustment for the sampling weight, stratification, the cluster sampling design, and the calculation of standard errors of the large survey data [31, 40]. We conducted tests for correlations among the types of IPV. IBM SPSS 26.0 was used for data preparation and the model parameter estimates were obtained in the statistical software Stata MP 16.1 using the restricted maximum likelihood method (REML). The level of significance was set at 0.05.

### 2.4. Ethical consideration

The study adhered to national and international ethical guidelines for biomedical research involving human subjects [41], including the Helsinki declaration. The study protocol was reviewed and approved by the Regional Committee for Medical and Health Research Ethics (Code number: 2016/967/REK sør-øst A) and the Norwegian Centre for Research Data (Code number: 48407) at the University of Oslo. Our team also requested permission and access to the data from the CSA in Ethiopia and Inner City Fund (ICF) international by registering online on the website www.dhsprogram.com [42] and submitting the study protocol by highlighting the objectives of the study as part of the online registration process, (See, S1 File). The ICF Macro Inc, removed all information that could be used to identify the respondents; hence, anonymity of the data was maintained.

## 3. Results

### 3.1. Characteristics of participants

**Fig 1:**
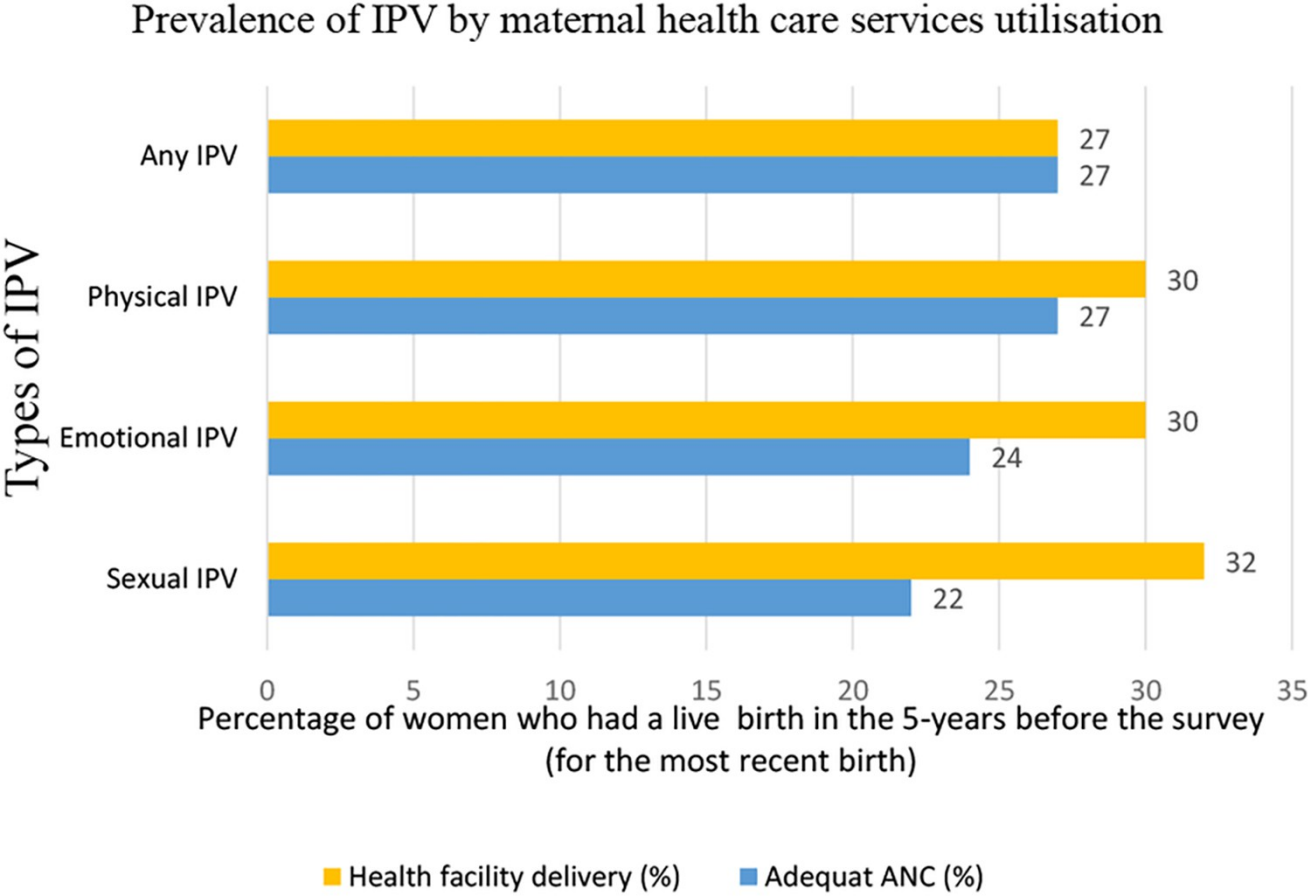
Prevalence of maternal healthcare service use among women’s who reported exposure to intimate partner violence in Ethiopia (2016, EDHS).

The mean age of all respondents was 29.1 (SD±6.6) years. The prevalence of lifetime exposure to emotional and physical IPV among mothers were nearly 23.0% each, while 9.4% of the study participants reported encountering sexual IPV. The overall lifetime prevalence of any forms of intimate partner violence (physical, sexual, and/or emotional) was 30.0%. The proportion of utilization of skilled maternal healthcare was relatively lower among women exposed to any violence. For instance, 23.6% of women who were exposed to emotional IPV had adequate uptake of ANC as compared to 32.2% of women not exposed to emotional IPV. Similarly, 22.4% of women who were exposed to sexual IPV had adequate uptake of ANC, vs. 31.0% of those not exposed to sexual IPV. The result was consistent when we examined differences in the uptake of maternal health care utilization further for any type of IPV (emotional, physical, and/ or sexual), (Fig 1).

There were moderate to strong correlation in magnitude between the three types of IPV (emotional, physical, and/or sexual); ranging from 0.3 to 0.5. Bivariate associations show considerable differences in the use of maternal healthcare services (adequate ANC visits, and health facility delivery) by socio-demographic background characteristics. The overall use of maternal healthcare services was higher among women in urban areas (59.4% for adequate ANC & 85.2% for health facility delivery) as compared to their rural counterparts (26.2% for adequate ANC & 27.0% for health facility delivery). The percentage of women receiving maternal healthcare services was lower among older women (35–49 years). Similarly, the percentage of women with adequate ANC (32.9%) and health facility delivery (36.8%) was higher among those respondents who had medium decision-making autonomy as compared to other sub-groups. Women who had exposure to mass media received a higher proportion of maternal healthcare services (49.6% for adequate ANC, & 62.9% for health facility delivery), as compared to those who had no mass media exposure. The result also showed that the utilization of maternal healthcare services differs considerably across geographical contextual regions. The usage of adequate ANC was highest was in the city dweller’s, while the lowest ANC utilization was observed in the pastoralist regions. The same differences is observed for health facility delivery, (Table 1).

**Table 1:** Sociodemographic characteristics and exposure to IPV by utilization of maternal healthcare services among (2836)^aa^ women in the 2016 Ethiopian Demographic Health Survey.

	Maternal Health Service Utilization			
Types of maternal healthcare	ANC		Place of delivery	
	Adequate ANC visits	Inadequate ANC visits^b^	Home	Health facility
Mean age (Year) (±SD)	28.7 (±6.2)	29.3 (±6.7)	29.6 (±6.7)	28.2 (±6.2)
**Intimate Partner Violence (IPV)**	856 (30.2%)	1980 (69.8%)	1871 (66.0%)	965 (34%)
**Emotional IPV**	***p***^a^ ***= 0*.*003***		*p = 0*.*148*	
Yes (23.1%)	23.6	76.4	69.7	30.3
No (76.9%)	32.2	67.8	64.9	35.1
**Physical IPV**	*p = 0*.*231*		*p = 0*.*107*	
Yes (23.4%)	27.4	72.6	69.9	30.1
No (76.6%)	31.0	69.0	64.8	35.2
**Sexual IPV**	***p = 0*.*051***		*p = 0*.*386*	
Yes (9.4%)	22.4	77.6	67.9	32.1
No (90.6%)	31.0	69.0	65.8	34.2
**Any form of IPV**	*p = 0*.*281*		***p = 0*.*033***	
Yes (29.8%)	27.5	72.5	72.5	27.5
No (70.2%)	31.3	68.7	63.2	36.8
**Individual level Factors**				
**Age** (years)	***p = 0*.*043***		***p = 0*.*000***	
15–24 (22.4%)	28.2	71.8	57.5	42.5
25–34 (51.7%)	33.1	66.9	66.5	33.5
35–49 (25.9%)	26.1	73.9	66.0	34.0
**Order of last birth**	***p = 0*.*000***		***p = 0*.*000***	
First (18.2%)	38.6	61.4	42.5	57.5
Second or third (30.2%)	34.5	65.5	59.2	40.8
Fourth or higher (51.6%)	24.7	75.3	78.2	21.8
**Education level of the women**	***p = 0*.*000***		***p = 0*.*000***	
No education (64.2%)	23.1	76.9	77.3	22.7
Primary and above (35.8%)	42.9	57.1	45.7	54.3
**Education level of their partners**	***p = 0*.*000***		***p = 0*.*000***	
No education (48.4%)	21.7	78.3	76.8	23.2
Primary and above (51.6%)	38.4	61.6	55.8	44.2
**Household wealth index**	***p = 0*.*000***		***p = 0*.*000***	
Low household wealth status (43.1%)	20.4	79.6	79.1	20.9
Medium household status (22.4%)	31.2	68.8	29.8	70.2
High household Wealth status (34.5%)	41.7	58.3	46.9	53.1
**Exposure to mass media**	***p = 0*.*000***		***p = 0*.*000***	
No exposure (67.2%)	24.1	75.9	74.4	25.6
Exposed to either radio or TV (19.2%)	37.9	62.1	56.9	43.1
Exposed to both radio and TV (13.5%)	49.6	50.4	37.1	62.9
**Relationship Factors**				
**Attitude towards Wife Beating**	***p = 0*.*007***		*p = 0*.*084*	
Accepts violence (fully) (69.0%)	35.4	64.6	61.6	38.4
Rejects violence (31.0%)	27.6	72.4	68.2	31.8
**Decision-making Autonomy** ^c^	***p = 0*.*000***		***p = 0*.*001***	
No autonomy (10.0%)	12.8	87.2	80.6	19.4
Medium autonomy (22.0%)	32.9	76.1	63.2	36.8
High autonomy (68.1%)	32.1	67.9	64.6	35.4
**Contextual community level factors**				
**Place of residence**	***p = 0*.*000***		***p = 0*.*000***	
Urban (12.1%)	59.4	40.6	14.8	85.2
Rural (87.9%)	26.2	73.8	73.0	27.0
**Contextual Regions**	***p = 0*.*000***		***p = 0*.*000***	
Agrarian (91.4%)	29.2	70.8	66.9	33.1
Pastoralist (5.8%)	22.6	77.4	77.8	22.2
City dweller’s (2.8%)	77.6	22.4	12.0	88.0

Note: a) *P* refers to a *p*-value of the Chi-squared test(X^2^); b) Inadequate—less than four ANC visits; Adequate & Inadequate ANC visits are additive. c) Total figure may not add to 100 percent due to “do not know” and “missing cases; ^aa^Weighted sample size.

### 3.2. Association between maternal exposure to IPV and use of maternal healthcare services

The association between maternal exposure to emotional IPV and the use of adequate ANC was statistically significant (**OR = 0.73, (95% CI:0.56–0.95**)), after controlling for significant confounders. Although, not statistically significant women who experienced sexual IPV were 1.42 times more likely to have health facility delivery than their counterparts who did not report such IPV, accounting for the influence of other covariates. We found no significant association between emotional, physical, sexual and/or any IPV and the utilization of health facility delivery. We obtained an ICC of 0.26 for ANC and 0.44 for health facility delivery from the adjusted multilevel logistic regression. This implies that the differences between the clusters account for 24% of the variability in the distribution of women with uptake of adequate ANC visits and 44% of the variability in the distribution of facility delivery, (Table 2). The best-fit model was determined using the lowest AIC. The results of the complete regression for all determinants by each type of IPV and outcome variables are available: (See S2 Table).

**Table 2:** Multiple multilevel logistic regression for association between maternal ever exposure to different forms of IPV and use of maternal healthcare services for currently married women.

	ANC Visits & Place of delivery (weighted)	
	Adequate ANC visits	Health facility delivery
	AOR (95% CI)	AOR (95% CI)
**Emotional IPV**		
No (ref) (76.9%)	1 (1,1)	1 (1,1)
Yes (23.1%)	0.73 (0.56, 0.95)**	1.16 (0.86, 1.57)
**Physical IPV**		
No (ref) (76.6%)	1 (1,1)	1 (1,1)
Yes (23.4%)	0.95 (0.74, 1.23)	0.88 (0.66, 1.18)
**Sexual IPV**		
No (ref) (90.6%)	1 (1,1)	1 (1,1)
Yes (9.4%)	0.68 (0.46, 1.01)	1.42 (0.93, 2.17)
**Any IPV**		
No (ref) (70.2%)	1 (1,1)	1 (1,1)
Yes (29.8%)	0.78 (0.54, 1.14)	0.60 (0.36, 1.00)
Intraclass Correlation Coefficient	0.26	0.44
Akaike Information Criterion	2793.60	2370.00

Note: sig. at **sig. at 5% level; ref = reference group; CI = Confidence Interval; IPV = Intimate Partner Violence; ANC = Antenatal Care; AOR = Adjusted Odds ratios. Models adjusted for: Mother’s age, birth order, mother’s education, husband’s education, wealth status, media exposure, decision making autonomy, place of residence, and contextual regions.

### 3.3. Moderation effects

To assess whether women’s education and/or household wealth moderated the effects of spousal IPV on maternal health outcomes, wealth/education and IPV interaction terms (wealth/education x IPV) were analysed. Overall, in the multivariate logistic moderation analysis, three moderation effects were identified. The first effect was the association between exposure to physical IPV and ANC, which was moderated by high wealth index (**AOR = 0.48, (95% CI:0.27–0.87)**, ***p = 0*.*016***), after controlling for potential confounders. The second was that women’s education positively moderates the relationship between any forms of IPV and ANC service utilization, (**AOR = 1.81, (95% CI:1.08–3.06), *p = 0*.*025*)**. The third effects was in the relationship between exposure to sexual IPV and utilization of health facility delivery, medium household wealth moderates the association significantly (**AOR = 0.27, (95% CI: 0.09–0.78), *p = 0*.*016*),** (Tables 3 & 4). In the stratified analysis, physical IPV was associated with uptake of adequate ANC visit in women in the rich wealth index, (***AOR = 0*.*61*, *(95% CI*:*0*.*37–0*.*98));*** the respective AOR (CI) in those with medium and low wealth index were: (***AOR = 1*.*06*, *(95% CI*:*0*.*53–2*.*12)) and*** (***AOR = 1*.*14*, *(95% CI*:*0*.*72–1*.*79))*.** In addition, a significant association between sexual IPV and health facility delivery was found for women in high wealth index (***AOR = 2*.*96*, *(95% CI*:*1*.*02–8*.*61)*,** albeit wide confidence intervals; no such associations were detected in the other wealth groups. There was an association between exposure to any forms of IPV and uptake of adequate ANC visit for those with no education (***AOR = 0*.*50*, *(95% CI*:*0*.*28–0*.*87);*** the association was in a different direction but none significant for those with primary or above education AOR = 1.25 (95% CI: 0.70, 2.23), (see S3 Table).

**Table 3:** Association between ever exposure to physical/any forms of IPV, and utilization of antenatal care and the moderating effect of education and wealth.

	Adequate ANC Visits N = 2863 (weighted)					
education x physical IPV Model				wealth x physical IPV Model		
Characteristics	AOR (95% CI)	*P-value*	Characteristics		AOR (95% CI)	*P-value*
**Physical IPV and Education level of the women** (interaction term)			**Physical IPV and wealth index** (interaction term)			
Yes X Primary and above	1.29 (0.76, 2.18)	*p = 0*.*343*	Yes X Medium wealth index		0.75 (0.40, 1.40)	*p = 0*.*365*
			Yes X High wealth index		**0.48 (0.27, 0.87)**	*p = 0*.016**
**Any IPV and Education level of the women** (interaction term)			**Any IPV and wealth index** (interaction term)			
Yes X Primary and above	**1.81 (1.08, 3.06)**	*0*.025**	Yes X Medium wealth index		1.40 (0.72, 2.72)	*p = 0*.*314*
			Yes X High wealth index		1.56 (0.83, 2.94)	*p = 0*.*168*
Coefficient Akaike Information Criterion	2800.18				2797.20	

Note: **sig. at 5% level; CI = Confidence Interval; ANC = Antenatal Care; AOR = Adjusted Odds ratios. Models adjusted for: Mother’s age, birth order, mother’s and husband’s education or household wealth index, media exposure, decision making autonomy, place of residence, and contextual regions.

**Table 4:** Association between ever exposure to Sexual IPV, and utilization of health facility delivery and the moderating effect of education and wealth.

	Health Facility delivery N = 2863 (weighted)				
education x sexual IPV Model			wealth x sexual IPV Model		
Characteristics	AOR (95% CI)	*P-value*	Characteristics	AOR (95% CI)	*P-value*
**Sexual IPV and Education level of the women** (interaction term)			**Sexual IPV and wealth index** (interaction term)		
Yes X Primary and above	0.81 (0.34, 1.94)	*p = 0*.*641*	Yes X Medium wealth index	**0.27 (0.09, 0.78)**	*p = 0*.016**
			Yes X High wealth index	1.45 (0.52, 4.10)	*p = 0*.*478*
Coefficient Akaike Information Criterion	**2372.82**			**2366.40**	

Note

**sig. at 5% level; CI = Confidence Interval; ANC = Antenatal Care; AOR = Adjusted Odds ratios. Models adjusted for: Mother’s age, birth order, mother’s and husband’s education or household wealth index, media exposure, decision making autonomy, place of residence, and contextual regions.

#### 3.3.1. Average marginal effects

We observed two different effects of physical IPV on ANC use: the effect of exposure to physical IPV on ANC is significant in the negative direction among those classifying as rich households. Maternal exposure to physical IPV makes a difference in ANC probability with a 7% points lower in the highest wealth sub-group, and about 4% points increase in the low wealth sub-group. The effect of exposure to sexual IPV on health facility delivery is significant in the positive direction among those rich households. Maternal exposure to sexual IPV variable makes a difference in health facility delivery probability of 14% points higher in the highest wealth sub-group, about 9% points lower in medium, and about 8% points increase in the poor sub-group. In addition, the effects of exposure to any form of IPV on ANC is significant in the negative direction among those with no education. Maternal exposure to any form of IPV variable makes a difference in ANC probability of approximately 2% points increase in the primary or above education group, and about 7% points lower in the no-education group, (See, S4 Table).

## 4. Discussion

In this paper, we examine the relative roles of different types of IPV, and the uptake of maternal healthcare services in Ethiopia. The observed associations between maternal exposure to emotional IPV and the low uptake of ANC services are in concert with international studies [9, 43, 44]. Emotional IPV affects a woman’s health; compromising pursuit of appropriate maternal healthcare [10]. Women exposed to any forms of IPV were at a higher risk for decreased use of maternal healthcare services than those not exposed to IPV. The increased risk of IPV exposures in impending utilization of maternal healthcare services underline the importance of addressing all forms of IPV when seeking to increase skilled maternal healthcare services utilization. The finding revealed no significant association between specific forms of IPV (emotional, physical and/or sexual IPV) and utilization of health facility delivery. This is consistent with a statewide survey in Malawi [45]. The results may partly underline the effectiveness of the Ethiopian health extension workers in timely identification of pregnancies and encouraging to access health facility services closer to their communities [46]. Therefore, regardless of whether women may experience IPV in these settings, they still manage skilled delivery since these initiatives make the services easily accessible. As reported in the result section, the presence of moderate to strong positive correlations between the three types of IPV (physical, sexual, and/or emotional IPV), might have indicated that those with exposure to physical IPV also reported high levels of emotional IPV and less likely to utilize maternal healthcare services.

To the best of our knowledge, this is the first Ethiopian population based study exploring any moderation effect of women’s educational attainment or household wealth status on the adverse impact IPV has on maternal healthcare service utilization. The association between any form of IPV and the use of adequate ANC service was moderated by women’s level of education; accordingly, women with lower education were less likely to visit adequate ANC services compared to those who had primary or above education. Education and household wealth are protective factors when exposed to domestic violence [28], increasing the likelihood of antenatal care and health facility delivery utilization in this setting. This study demonstrated that women who exposed to physical IPV were less likely to attend adequate ANC services when women belong to rich households. Previous studies have shown that economically empowered women may be exposed to IPV by their partners who feel threatened by their wealth [47, 48]. However, most studies report improved socioeconomic status as a protective factor against IPV [33, 49–51]. International studies are in concert with our study and emphasize the high risk of poor uptake of skilled maternal healthcare among IPV exposed women with low education [52–55]. Socio-economic (education and wealth) circumstances also affects observed associations between IPV and maternal healthcare service utilization, as seen in the education/wealth-stratified analyses of our study.

Maternal healthcare utilization in Ethiopia in general has been lower than other countries [56]. The factors affecting uptake of services are many: education, empowerment, economic attainment and IPV affect all aspect of women’s lives. This is compounded in Ethiopia by cultural diversity, institutionalized gender roles and structural power imbalances between women and men [57]. The social inequalities can increase the risk as well as the overall impact of IPV, constraining women from access to maternal healthcare services [4]. This is highlights the importance to consider IPV when planning and implementing maternal health programs in the Ethiopian society. Given the high prevalence of IPV in Ethiopia, focused efforts are required by government and other stakeholders to change the cultural, structural, institutional and social barriers to mitigate impact and reduce occurrence of IPV. The perspective of community stakeholders and healthcare providers related to IPV underscore these challenges [58, 59]. Taking into consideration other recent studies addressing the effect of IPV on maternal health and healthcare utilization [25, 60], it is evident that in order to reach several of the health care and political goals set in Ethiopia, IPV prevention has to be high on the agenda. It is critical to integrate IPV prevention and intervention efforts within the existing maternal and child health programmes to address the needs of abused pregnant women and contribute to change in maternity care in Ethiopia and the achievements of the sustainable development goals.

### 4.1. Study strength and limitations

This study used nationally representative weighted data so the findings can be generalizable to the reproductive-aged women in Ethiopia. However, the wide confidence intervals indicate a challenge with the sample size for some analyses. Our reliance on cross-sectional data and the retrospective nature of the study limits the ability to draw causal inferences between exposure to IPV and the outcomes. Women in the current survey may not have felt comfortable to disclose exposure of IPV during pregnancy, and the data collection may be subject to recall or response bias. The study assessed lifetime IPV and the use of maternal healthcare services rather than IPV within the last 12 months. Another factor might be the contextual acceptance of sexual IPV as it is defined in the data collection tools used in the current survey. The cultural appropriateness of the international data collection tools used in the DHS needs further study in Ethiopia.

## 5. Conclusion

Exposure to emotional IPV was associated with poor uptake of adequate ANC service for married Ethiopian women. The moderation effects need to be taken into consideration when planning interventions. These interventions should target women with low education and wealth status. Various strategies related to increased education and economical security in women especially with the intersection of IPV should be studied.

## Supporting information

S1 File Approval letter for data access. (PDF)

S1 Table Univariate logistic regression. (DOCX)

S2 Table The full results of regression analysis. (DOCX)

S3 Table Stratified adjusted logistic regression model. (DOCX)

S4 Table Average marginal effects. (DOCX)
