# Supplementary material for: Maternal exposure to intimate partner violence and uptake of maternal healthcare services in Ethiopia: Evidence from a national survey
Source: PLoS One. 2022 Aug 18;17(8):e0273146. doi: 10.1371/journal.pone.0273146 (PMC9387817; doi:10.1371/journal.pone.0273146)
Supplement: S1 File — (PDF) [file pone.0273146.s001.pdf]

በኢትዮጲያ ፌዴራላዊ ዲሞክራሲያዊ ሪፐብሊክ
በብሔራዊ የፕላን ኮሚሽን
የማዕከላዊ ስታቲስቲክስ ኤጀንሲ

THE FEDERAL DEMOCRATIC REPUBLIC OF ETHIOPIA
NATIONAL PLANNING COMMISSION
CENTRAL STATISTICAL AGENCY

ቁጥር 3-15/3578
Ref. NO. 08 SEP 2017
ቀን
Date

To: **University of Oslo, Faculty of Medicine, Norway**

Subject: **Approval letter for data Access**

SemanKedirOusman requested our organization to write a letter of approval inorder to use the Ethiopian Demographic and Health Survey (EDHS) raw data for the year 2000, 2005, 2011, and 2016 for third degree fulfillment.

Therefore, the Centeral Statistical Agency of Ethiopia (CSA) will authorize to use the after mentioned raw data for his doctoral project purpose based on the raw data access police of the organization. Accordingly, the requested data should only be used for the purpose of the registered research or study.

With regards

[illegible]

The Federal Democratic Republic of Ethiopia National Planning Commission Central Statistical Agency

+251-1155-3011/ +251-1156-3882/ +251-1111-5131/
+251-1155-3112/ +251-1157-3296
ፋክስ ቁ. Fax No. +251-1111-5470/ +251-1155-334
ድረ ገጽ፦ Website: www.csa.gov.et
1143
አዲስ አበባ - ኢትዮጵያ
Addis Ababa - Ethiopia
