# Supplementary material for: Maternal exposure to intimate partner violence and uptake of maternal healthcare services in Ethiopia: Evidence from a national survey
Source: PLoS One. 2022 Aug 18;17(8):e0273146. doi: 10.1371/journal.pone.0273146 (PMC9387817; doi:10.1371/journal.pone.0273146)
Supplement: S1 Table — (DOCX) [file pone.0273146.s002.docx]

Additional File Table S1. Univariate logistic regression for association between maternal exposure to different forms of IPV and use of maternal healthcare services for 2863 (weighted) currently married Ethiopian women using the 2016 Ethiopian Demographic Health Survey.

| EDHS Years | **2016** |  |
| --- | --- | --- |
| Maternal health outcomes | **Adequate ANC Visits** | **Institutional Delivery** |
| Models | **Model I** | **Model II** |
| **Characteristics** | **COR (95 % CI)** | **COR (95 % CI)** |
| **Individual level Factors** |  |  |
| **Violence related** |  |  |
| **Emotional IPV** |  |  |
| No (ref) | 1 (1,1) | 1 (1,1) |
| Yes | **0.67(0.52, 0.85)***** | 0.93 (0.71, 1.20) |
| **Physical IPV** |  |  |
| No (ref) | 1 (1,1) | 1 (1,1) |
| Yes | 1.04 (0.82, 1.32) | 0.86 (0.66, 1.12) |
| **Sexual IPV** |  |  |
| No (ref) | 1 (1,1) | 1 (1,1) |
| Yes | 0.72 (0.50, 1.03) | 1.21 (0.82 1.77) |
| **Any form of IPV** |  |  |
| No (ref) | 1 (1,1) | 1 (1,1) |
| Yes | 0.74 (0.55, 1.01) | **0.64 (0.44, 0.92)**** |
| **Age when giving last birth** (years) |  |  |
| 15—24 (ref) | 1 (1,1) | 1 (1,1) |
| 25—34 | 1.19 (0.92, 1.53) | **0.46 (0.34, 0.60)***** |
| 35—49 | 0.81 (0.60, 1.09) | **0.34 (0.25, 0.48)***** |
| **Order of the last birth** |  |  |
| First (ref) | 1 (1,1) | 1 (1,1) |
| Second or third | 0.77 (0.59, 1.02) | **0.35 (0.25, 0.48)***** |
| Fourth or higher | **0.47 (0.37, 0.64)***** | **0.15 (0.11, 0.21)***** |
| **Education level of the women** |  |  |
| No education (ref) | 1 (1,1) | 1 (1,1) |
| Primary and above | **2.66 (2.15, 3.29)***** | **4.06 (3.19, 5.17)***** |
| **Education level of their partners** |  |  |
| No education (ref) | 1 (1,1) | 1 (1,1) |
| Primary and above | **2.53 (2.02, 3.16)***** | **2.70 (2.10, 3.48)***** |
| **Household wealth index** |  |  |
| Low household wealth status (ref) | 1 (1,1) | 1 (1,1) |
| Middle | **1.71 (1.32, 2.23)***** | **1.65 (1.24, 2.20)***** |
| High household Wealth status | **2.57 (2.00, 3.31)***** | **3.77 (2.84, 5.01)***** |
| **Exposure to mass media** |  |  |
| No exposure (ref) | 1 (1,1) | 1 (1,1) |
| Exposed to either radio or TV | **1.70 (1.32, 2.18)***** | **1.70 (1.28, 2.27)***** |
| Exposed to both radio and TV | **3.01 (2.23, 4.05)***** | **6.10 (4.23, 8.79)***** |
| **Relationship Factors** |  |  |
| **Attitude towards Wife Beating** |  |  |
| Rejects violence (ref) | 1 (1,1) | 1 (1,1) |
| Accepts violence (fully) | 0.59 (0.48, 0.72) | 0.52 (0.41, 0.65) |
| **Decision-making Autonomy** |  |  |
| No autonomy (ref) | 1 (1,1) | 1 (1,1) |
| Medium autonomy | **3.63 (2.26, 5.82)***** | **2.95 (1.83, 4.77)***** |
| High autonomy | **4.08 (2.62, 6.37)***** | **2.54 (1.63, 3.95)***** |
| **Contextual community level factors** |  |  |
| **Place of residence** |  |  |
| Urban (ref) | 1 (1,1) | 1 (1,1) |
| Rural | **0.15 (0.10, 0.22)***** | **0.02 (0.01, 0.04)***** |
| **Contextual Regions** |  |  |
| Agrarian (ref) | 1 (1,1) | 1 (1,1) |
| Pastoralist | **0.58 (0.35, 0.95)**** | **0.36 (0.20, 0.67)***** |
| City dweller’s | **10.47 (5.30, 20.70)***** | **28.42 (10.95, 73.75)***** |

Note: sig. at **sig. at 5% level; ***sig. at 1% level; ref = reference group; CI = Confidence Interval; IPV = Intimate Partner Violence; ANC = Antenatal Care; COR = Crude Odds Ratios.
