# Supplementary material for: Maternal exposure to intimate partner violence and uptake of maternal healthcare services in Ethiopia: Evidence from a national survey
Source: PLoS One. 2022 Aug 18;17(8):e0273146. doi: 10.1371/journal.pone.0273146 (PMC9387817; doi:10.1371/journal.pone.0273146)
Supplement: S2 Table — (DOCX) [file pone.0273146.s003.docx]

Additional File Table S3.

Table 3a. Multiple multilevel logistic regression for association between maternal exposure to **emotional** IPV and use of maternal healthcare services for 2863 (weighted) currently married Ethiopian women using the 2016 Ethiopian Demographic Health Survey.

|  | **ANC Visits & Place of delivery** | | |  |
| --- | --- | --- | --- | --- |
|  | **Adequate ANC visits** |  | **Health facility** |  |
|  | **AOR (95 % CI)** | ***P*-value** | **AOR (95 % CI)** | ***P*-value** |
| **Emotional IPV** |  |  |  |  |
| No (ref) (76.9%) | 1 (1,1) |  | 1 (1,1) |  |
| Yes (23.1%) | **0.73 (0.56, 0.95)** | ***0.019***** | 1.16 (0.86, 1.57) | *0.323* |
| **Age** (years) |  |  |  |  |
| 15—24 (ref) | 1 (1,1) |  | 1 (1,1) |  |
| 25—34 | **1.69 (1.20, 2.36)** | ***0.002****** | 1.07 (0.73, 1.57) | *0.709* |
| 35—49 | **1.79 (1.16, 2.77)** | ***0.008****** | 1.37 (0.83, 2.24) | *0.215* |
| **Order of last birth** |  |  |  |  |
| First (ref) | 1 (1,1) |  | 1 (1,1) |  |
| Second or third | 0.89 (0.63, 1.25) | *0.499* | **0.39 (0.26, 0.58)** | ***0.000****** |
| Fourth or higher | **0.66 (0.45, 0.98)** | ***0.041***** | **0.20 (0.13, 0.32)** | ***0.000****** |
| **Education level of the women** |  |  |  |  |
| No education (ref) | 1 (1,1) |  | 1 (1,1) |  |
| Primary and above education | **1.77 (1.35, 2.32)** | ***0.000****** | **1.67 (1.23, 2.26)** | ***0.000****** |
| **Education level of their partners** |  |  |  |  |
| No education (ref) | 1 (1,1) |  | 1 (1,1) |  |
| Primary and above | **1.75 (1.37, 2.25)** | ***0.000****** | 1.26 (0.95, 1.68) | *0.215* |
| **Exposure to mass media** |  |  |  |  |
| No exposure (ref) | 1 (1,1) |  | 1 (1,1) |  |
| Exposed to either radio or TV (19.2%) | 1.18 (0.89, 1.56) | *0.255* | 0.97 (0.70, 1.34) | *0.851* |
| Exposed to both radio and TV (13.5%) | **1.46 (1.02, 2.07)** | ***0.036***** | **2.68 (1.74, 4.12)** | ***0.000****** |
| **Household wealth index** |  |  |  |  |
| Low household wealth status (ref) | 1 (1,1) |  | 1 (1,1) |  |
| Medium | **1.55 (1.17, 2.05)** | ***0.002****** | **1.38 (1.01, 1.88)** | ***0.046***** |
| High household Wealth status | 1.05 (0.77, 1.42) | *0.765* | **1.45 (1.03, 2.04)** | ***0.035***** |
| **Decision-making Autonomy**^c^ |  |  |  |  |
| No autonomy (ref) | 1 (1,1) |  | 1 (1,1) |  |
| Medium autonomy | **3.09 (1.92, 4.97)** | ***0.000****** | **2.53 (1.54, 4.15)** | ***0.000****** |
| High autonomy | **3.28 (2.09, 5.14)** | ***0.000****** | **1.92 (1.22, 3.04)** | ***0.005****** |
| **Place of residence** |  |  |  |  |
| Urban (ref) | 1 (1,1) |  | 1 (1,1) |  |
| Rural | **0.40 (0.24, 0.67)** | ***0.000****** | **0.05 (0.02, 0.11)** | ***0.000****** |
| **Contextual Regions** |  |  |  |  |
| Agrarian (ref) | 1 (1,1) |  | 1 (1,1) |  |
| Pastoralist | 0.79 (0.47, 1.33) | *0.378* | **0.38 (0.19, 0.74)** | ***0.004****** |
| City dweller’s | **2.80 (1.28, 6.12)** | ***0.010***** | 1.98 (0.58, 6.72) | *0.274* |
| Intraclass correlation | **0.26** |  | **0.44** |  |
| Coefficient Akaike Information Criterion | **2793.60** |  | **2373.00** |  |

Note: sig. at *sig.at 10% level; **sig. at 5% level; ***sig. at 1% level; ref = reference group; CI = Confidence Interval; n = Number of participants; IPV = Intimate Partner Violence; ANC = Antenatal Care; AOR = Adjusted Odds ratios. Models adjusted for: Mother’s age, birth order, mother & husband’s education, media exposure, household wealth status, decision making autonomy, place of residence, and contextual regions.

Table 3b. Multiple multilevel logistic regression for association between maternal exposure to **Physical** IPV and use of maternal healthcare services for 2863 (weighted) currently married Ethiopian women using the 2016 Ethiopian Demographic Health Survey.

|  | **ANC Visits & Place of delivery** | | |  |
| --- | --- | --- | --- | --- |
|  | **Adequate ANC visits** |  | **Health facility** |  |
|  | **AOR (95 % CI)** | ***P*-value** | **AOR (95 % CI)** | ***P*-value** |
| **Physical IPV** |  |  |  |  |
| No (ref) (76.9%) | 1 (1,1) |  | 1 (1,1) |  |
| Yes (23.1%) | 0.95 (0.74, 1.23) | *0.716* | 0.88 (0.66, 1.18) | *0.403* |
| **Age** (years) |  |  |  |  |
| 15—24 (ref) | 1 (1,1) |  | 1 (1,1) |  |
| 25—34 | **1.68 (1.20, 2.36)** | ***0.002****** | 1.07 (0.73, 1.56) | *0.724* |
| 35—49 | **1.76 (1.14, 2.71)** | ***0.011***** | 1.39 (0.85, 2.28) | *0.190* |
| **Order of last birth** |  |  |  |  |
| First (ref) | 1 (1,1) |  | 1 (1,1) |  |
| Second or third | 0.88 (0.63, 1.23) | *0.452* | **0.40 (0.27, 0.59)** | ***0.000****** |
| Fourth or higher | **0.65 (0.44, 0.96)** | ***0.031***** | **0.20 (0.13, 0.32)** | ***0.000****** |
| **Education level of the women** |  |  |  |  |
| No education (ref) | 1 (1,1) |  | 1 (1,1) |  |
| Primary and above education | **1.76 (1.34, 2.30)** | ***0.000****** | **1.69 (1.25, 2.28)** | ***0.001****** |
| **Education level of their partners** |  |  |  |  |
| No education (ref) | 1 (1,1) |  | 1 (1,1) |  |
| Primary and above | **1.74 (1.36, 2.23)** | ***0.000****** | 1.26 (0.94, 1.67) | *0.116* |
| **Exposure to mass media** |  |  |  |  |
| No exposure (ref) | 1 (1,1) |  | 1 (1,1) |  |
| Exposed to either radio or TV (19.2%) | 1.19 (0.90, 1.58) | *0.224* | 0.97 (0.70, 1.35) | *0.876* |
| Exposed to both radio and TV (13.5%) | **1.50 (1.06, 2.14)** | ***0.023***** | **2.65 (1.73, 4.07)** | ***0.000****** |
| **Household wealth index** |  |  |  |  |
| Low household wealth status (ref) | 1 (1,1) |  | 1 (1,1) |  |
| Medium | **1.57 (1.18, 2.07)** | ***0.002****** | **1.36 (0.99, 1.86)** | ***0.054***** |
| High household Wealth status | 1.06 (0.78, 1.44) | *0.703* | **1.42 (1.01, 2.00)** | ***0.044***** |
| **Decision-making Autonomy**^c^ |  |  |  |  |
| No autonomy (ref) | 1 (1,1) |  | 1 (1,1) |  |
| Medium autonomy | **3.17 (1.97, 5.10)** | ***0.000****** | **2.51 (1.53, 4.12)** | ***0.000****** |
| High autonomy | **3.38 (2.16, 5.29)** | ***0.000****** | **1.90 (1.20, 2.99)** | ***0.006****** |
| **Place of residence** |  |  |  |  |
| Urban (ref) | 1 (1,1) |  | 1 (1,1) |  |
| Rural | **0.40 (0.24, 0.67)** | ***0.000****** | **0.05 (0.02, 0.11)** | ***0.000****** |
| **Contextual Regions** |  |  |  |  |
| Agrarian (ref) | 1 (1,1) |  | 1 (1,1) |  |
| Pastoralist | 0.81 (0.48, 1.36) | *0.429* | **0.37 (0.19, 0.72)** | ***0.004****** |
| City dweller’s | **2.73 (1.25, 5.98)** | ***0.012***** | 2.02 (0.60, 6.82) | *0.259* |
| Intraclass correlation | **0.26** |  | **0.44** |  |
| Coefficient Akaike Information Criterion | **2799.00** |  | **2373.00** |  |

Table 3c. Multiple multilevel logistic regression for association between maternal exposure to **Sexual** IPV and use of maternal healthcare services for 2863 (weighted) currently married Ethiopian women using the 2016 Ethiopian Demographic Health Survey.

|  | **ANC Visits & Place of delivery** | | |  |
| --- | --- | --- | --- | --- |
|  | **Adequate ANC visits** |  | **Health facility** |  |
|  | **AOR (95 % CI)** | ***P*-value** | **AOR (95 % CI)** | ***P*-value** |
| **Sexual IPV** |  |  |  |  |
| No (ref) (76.9%) | 1 (1,1) |  | 1 (1,1) |  |
| Yes (23.1%) | 0.68 (0.46, 1.01) | *0.058* | 1.42 (0.93, 2.17) | *0.104* |
| **Age** (years) |  |  |  |  |
| 15—24 (ref) | 1 (1,1) |  | 1 (1,1) |  |
| 25—34 | **1.70 (1.21, 2.38)** | ***0.002****** | 1.07 (0.73, 1.56) | *0.739* |
| 35—49 | **1.78 (1.15, 2.74)** | ***0.009****** | 1.36 (0.83, 2.24) | *0.217* |
| **Order of last birth** |  |  |  |  |
| First (ref) | 1 (1,1) |  | 1 (1,1) |  |
| Second or third | 0.88 (0.63, 1.23) | *0.459* | **0.40 (0.27, 0.59)** | ***0.000****** |
| Fourth or higher | **0.65 (0.44, 0.96)** | ***0.032***** | **0.20 (0.13, 0.32)** | ***0.000****** |
| **Education level of the women** |  |  |  |  |
| No education (ref) | 1 (1,1) |  | 1 (1,1) |  |
| Primary and above education | **1.76 (1.34, 2.30)** | ***0.000****** | **1.68 (1.24, 2.27)** | ***0.001****** |
| **Education level of their partners** |  |  |  |  |
| No education (ref) | 1 (1,1) |  | 1 (1,1) |  |
| Primary and above | **1.75 (1.36, 2.25)** | ***0.000****** | 1.25 (0.94, 1.67) | *0.122* |
| **Exposure to mass media** |  |  |  |  |
| No exposure (ref) | 1 (1,1) |  | 1 (1,1) |  |
| Exposed to either radio or TV (19.2%) | 1.19 (0.90, 1.58) | *0.221* | 0.96 (0.69, 1.34) | *0.833* |
| Exposed to both radio and TV (13.5%) | **1.51 (1.06, 2.15)** | ***0.022***** | **2.63 (1.71, 4.05)** | ***0.000****** |
| **Household wealth index** |  |  |  |  |
| Low household wealth status (ref) | 1 (1,1) |  | 1 (1,1) |  |
| Medium | **1.55 (1.17, 2.06)** | ***0.002****** | **1.38 (1.01, 1.89)** | ***0.044***** |
| High household Wealth status | 1.04 (0.77, 1.42) | *0.774* | **1.46 (1.03, 2.05)** | ***0.032***** |
| **Decision-making Autonomy**^c^ |  |  |  |  |
| No autonomy (ref) | 1 (1,1) |  | 1 (1,1) |  |
| Medium autonomy | **3.19 (1.98, 5.14)** | ***0.000****** | **2.50 (1.52, 4.09)** | ***0.000****** |
| High autonomy | **3.38 (2.16, 5.30)** | ***0.000****** | **1.90 (1.21, 3.00)** | ***0.006****** |
| **Place of residence** |  |  |  |  |
| Urban (ref) | 1 (1,1) |  | 1 (1,1) |  |
| Rural | **0.41 (0.24, 0.67)** | ***0.000****** | **0.05 (0.02, 0.11)** | ***0.000****** |
| **Contextual Regions** |  |  |  |  |
| Agrarian (ref) | 1 (1,1) |  | 1 (1,1) |  |
| Pastoralist | 0.79 (0.47, 1.34) | *0.387* | **0.38 (0.20, 0.74)** | ***0.005****** |
| City dweller’s | **2.70 (1.23, 5.92)** | ***0.013***** | 2.00 (0.50, 6.77) | *0.267* |
| Intraclass correlation | **0.26** |  | **0.44** |  |
| Coefficient Akaike Information Criterion | **2796.00** |  | **2371.00** |  |

Table 3d. Multiple multilevel logistic regression for association between maternal exposure to **Any** **form of IPV** and use of maternal healthcare services for 2863 (weighted) currently married Ethiopian women using the 2016 Ethiopian Demographic Health Survey.

|  | **ANC Visits & Place of delivery** | | |  |
| --- | --- | --- | --- | --- |
|  | **Adequate ANC visits** |  | **Health facility** |  |
|  | **AOR (95 % CI)** | ***P*-value** | **AOR (95 % CI)** | ***P*-value** |
| **Any form of IPV** |  |  |  |  |
| No (ref) (76.9%) | 1 (1,1) |  | 1 (1,1) |  |
| Yes (23.1%) | 0.78 (0.54, 1.14) | *0.207* | **0.60 (0.36, 1.00)** | ***0.051**** |
| **Age** (years) |  |  |  |  |
| 15—24 (ref) | 1 (1,1) |  | 1 (1,1) |  |
| 25—34 | **1.66 (1.19, 2.33)** | ***0.003****** | 1.06 (0.72, 1.54) | *0.777* |
| 35—49 | **1.73 (1.12, 2.67)** | ***0.013***** | 1.36 (0.83, 2.22) | *0.226* |
| **Order of last birth** |  |  |  |  |
| First (ref) | 1 (1,1) |  | 1 (1,1) |  |
| Second or third | 0.88 (0.63, 1.24) | *0.461* | **0.40 (0.27, 0.59)** | ***0.000****** |
| Fourth or higher | **0.65 (0.44, 0.97)** | ***0.036***** | **0.21 (0.13, 0.33)** | ***0.000****** |
| **Education level of the women** |  |  |  |  |
| No education (ref) | 1 (1,1) |  | 1 (1,1) |  |
| Primary and above education | **1.76 (1.34, 2.30)** | ***0.000****** | **1.68 (1.24, 2.27)** | ***0.001****** |
| **Education level of their partners** |  |  |  |  |
| No education (ref) | 1 (1,1) |  | 1 (1,1) |  |
| Primary and above | **1.75 (1.36, 2.24)** | ***0.000****** | 1.26 (0.95, 1.68) | *0.109* |
| **Exposure to mass media** |  |  |  |  |
| No exposure (ref) | 1 (1,1) |  | 1 (1,1) |  |
| Exposed to either radio or TV (19.2%) | 0.19 (0.90, 1.58) | *0.227* | 0.97 (0.70, 1.34) | *0.841* |
| Exposed to both radio and TV (13.5%) | **1.51 (1.06, 2.14)** | ***0.023***** | **2.64 (1.72, 4.06)** | ***0.000****** |
| **Household wealth index** |  |  |  |  |
| Low household wealth status (ref) | 1 (1,1) |  | 1 (1,1) |  |
| Medium | **1.56 (1.18, 2.06)** | ***0.002****** | 1.35 (0.99, 1.85) | *0.058* |
| High household Wealth status | 1.06 (0.78, 1.43) | *0.723* | **1.42 (1.01, 2.01)** | ***0.042***** |
| **Decision-making Autonomy**^c^ |  |  |  |  |
| No autonomy (ref) | 1 (1,1) |  | 1 (1,1) |  |
| Medium autonomy | **3.19 (1.98, 5.13)** | ***0.000****** | **2.52 (1.54, 4.13)** | ***0.000****** |
| High autonomy | **3.39 (2.17, 5.32)** | ***0.000****** | **1.90 (1.21, 3.01)** | ***0.006****** |
| **Place of residence** |  |  |  |  |
| Urban (ref) | 1 (1,1) |  | 1 (1,1) |  |
| Rural | **0.40 (0.24, 0.67)** | ***0.000****** | **0.05 (0.02, 0.11)** | ***0.000****** |
| **Contextual Regions** |  |  |  |  |
| Agrarian (ref) | 1 (1,1) |  | 1 (1,1) |  |
| Pastoralist | 0.82 (0.48, 1.38) | *0.454* | **0.38 (0.20, 0.74)** | ***0.004****** |
| City dweller’s | **2.71 (1.24, 5.95)** | ***0.013***** | 2.03 (0.59, 6.90) | *0.258* |
| Intraclass correlation | **0.26** |  | **0.44** |  |
| Coefficient Akaike Information Criterion | **2798.00** |  | **2370.00** |  |

Table e. Multiple multilevel logistic regression for association between maternal ever exposure to different forms of IPV and use of maternal healthcare services for currently married women, (N= 2863) weighted.

|  | **ANC Visits & Place of delivery** | |
| --- | --- | --- |
|  | **Adequate ANC visits** | **Health facility delivery** |
|  | **AOR (95 % CI)** | **AOR (95 % CI)** |
| **Emotional IPV** |  |  |
| No (ref) (76.9%) | 1 (1,1) | 1 (1,1) |
| Yes (23.1%) | **0.73 (0.56, 0.95)**** | 1.16 (0.86, 1.57) |
| **Physical IPV** |  |  |
| No (ref) (76.6%) | 1 (1,1) | 1 (1,1) |
| Yes (23.4%) | 0.95 (0.74, 1.23) | 0.88 (0.66, 1.18) |
| **Sexual IPV** |  |  |
| No (ref) (90.6%) | 1 (1,1) | 1 (1,1) |
| Yes (9.4%) | 0.68 (0.46, 1.01) | 1.42 (0.93, 2.17) |
| **Any IPV** |  |  |
| No (ref) (90.6%) | 1 (1,1) | 1 (1,1) |
| Yes (9.4%) | 0.78 (0.54, 1.14) | 0.60 (0.36, 1.00)** |
| Intraclass Correlation Coefficient | 0.26 | 0.44 |
| Akaike Information Criterion | 2793.60 | 2370.00 |
