# Supplementary material for: Maternal exposure to intimate partner violence and uptake of maternal healthcare services in Ethiopia: Evidence from a national survey
Source: PLoS One. 2022 Aug 18;17(8):e0273146. doi: 10.1371/journal.pone.0273146 (PMC9387817; doi:10.1371/journal.pone.0273146)
Supplement: S3 Table — (DOCX) [file pone.0273146.s004.docx]

Additional file Table S4a. Multiple multilevel logistic regression exploring association between maternal exposure to Physical IPV and uptake of adequate ANC visits stratified by education or income status for 2863 currently married women: 2016 Ethiopia DHS.

| Maternal health outcome | **Adequate ANC Visits** | | | | | | | |
| --- | --- | --- | --- | --- | --- | --- | --- | --- |
| Stratified by | **Education status** | | | | **HH wealth status** | | | |
| Stratum | No education (n=1788) | | *Primary or above (n=1075)* | | Low HH wealth (n=1496) | Medium HH wealth (n=433) | *High HH wealth index (n=934)* | |
| **Violence type** | AOR (95 % CI) | ***P-value*** | AOR (95 % CI) | ***P-value*** | AOR (95 % CI) |  | AOR (95 % CI) | |
| **Physical IPV** |  |  |  |  |  |  |  | |
| No (ref) | 1 (1,1) |  | 1 (1,1) |  | 1 (1,1) | 1 (1,1) | 1 (1,1) | |
| Yes | 0.92 (0.64, 1.32) | *p = 0.663* | 0.97 (0.60, 1.56) | *p = 0.898* | 1.14 (0.72, 1.79) | 1.06 (0.53, 2.12) | **0.61 (0.37, 0.98)**** | |
| **Age** (years) |  |  |  |  |  |  |  |  |
| 15—24 (ref) | 1 (1,1) |  | 1 (1,1) |  | 1 (1,1) | 1 (1,1) | 1 (1,1) | |
| 25—34 | 1.24 (0.68, 2.29) | *p = 0.480* | **1.72 (1.06, 2.81)** | ***p = 0.029***** | **2.91 (1.57, 5.38)***** | 2.66 (0.89, 7.92) | 0.88 (0.50, 1.56) | |
| 35—49 | 1.29 (0.64, 2.59) | *p = 0.470* | **2.49 (1.14, 5.44)** | ***p = 0.021***** | **2.15 (1.00, 4.63)**** | **5.91 (1.37, 25.55)**** | 1.54 (0.72, 3.27) | |
| **Order of last birth** |  |  |  |  |  |  |  | |
| First (ref) | 1 (1,1) |  | 1 (1,1) |  | 1 (1,1) | 1 (1,1) | 1 (1,1) | |
| Second or third | 1.50 (0.72, 3.12) | ***p =*** *0.277* | 0.87 (0.55, 1.38) | *p = 0.558* | 1.10 (0.57, 2.13) | 1.22 (0.37, 3.95) | 0.77 (0.45, 1.32) | |
| Fourth or higher | 1.05 (0.49, 2.29) | *p = 0.891* | **0.55 (0.30, 1.01)** | ***p = 0.053***** | **0.43 (0.20, 0.92)**** | 1.00 (0.27, 3.78) | 0.63 (0.33, 1.20) | |
| **Education level of the women** |  |  |  |  |  |  |  | |
| No education (ref) |  |  |  |  | 1 (1,1) | 1 (1,1) | 1 (1,1) | |
| Primary and above education | - | ***-*** | - | *-* | 0.97 (0.58, 1.62) | **7.50 (3.15, 17.84)***** | **2.41 (1.51, 3.85)***** | |
| **Education level of their partners** | **-** | ***-*** | - | *-* |  |  |  | |
| No education (ref) | 1 (1,1) |  | 1 (1,1) |  | 1 (1,1) | 1 (1,1) | 1 (1,1) | |
| Primary and above | **1.58 (1.14, 2.19)** | ***p = 0.006****** | **2.22 (1.31, 3.74)** | ***p = 0.003****** | 0.78 (0.50, 1.21) | **3.70 (1.92, 7.13)***** | **2.27 (1.36, 3.78)***** | |
| **Exposure to mass media** |  |  |  |  |  |  |  | |
| No exposure (ref) | 1 (1,1) |  | 1 (1,1) |  | 1 (1,1) | 1 (1,1) | 1 (1,1) | |
| Exposed to either radio or TV (19.2%) | 1.21 (0.78, 1.86) | *p = 0.397* | 1.04 (0.65, 1.68) | *p = 0.857* | **1.89 (1.04, 3.43)**** | 1.24 (0.57, 2.72) | 0.85 (0.53, 1.36) | |
| Exposed to both radio and TV (13.5%) | **2.88 (1.59, 5.20)** | ***p = 0.000****** | 1.02 (0.59, 1.77) | *0.949* | 1.58 (0.67, 3.71) | 2.12 (0.62, 7.26) | 1.43 (0.87, 2.35) | |
| **Household wealth index** |  |  |  |  |  |  |  | |
| Low household wealth status (ref) | 1 (1,1) |  | 1 (1,1) |  | **-** | - |  | |
| Medium household Wealth status | 1.04 (0.72, 1.51) | ***p =*** *0.826* | **4.08 (2.24, 7.45)** | ***p = 0.000****** | **-** | - |  | |
| High household Wealth status | **0.55 (0.35, 0.86)** | ***p = 0.009****** | **2.84 (1.64, 4.94)** | ***p = 0.000****** | **-** | - | - | |
| **Decision-making Autonomy**^c^ |  |  |  |  |  |  |  | |
| No autonomy (ref) | 1 (1,1) |  | 1 (1,1) |  | 1 (1,1) | 1 (1,1) | 1 (1,1) | |
| Medium autonomy | **5.03 (2.67, 9.48)** | ***p = 0.000****** | 2.02 (0.82, 4.99) | *p = 0.128* | **2.92 (1.34, 6.36)***** | **5.58 (1.66, 18.72)***** | **4.66 (1.41, 15.40)**** | |
| High autonomy | **3.89 (2.16, 7.02)** | ***p = 0.000****** | **3.25 (1.38, 7.63)** | ***p = 0.007****** | **2.82 (1.37, 5.82)***** | **4.87 (1.65, 14.38)***** | **4.93 (1.55, 15.71)***** | |
| **Place of residence** |  |  |  |  |  |  |  | |
| Urban (ref) | 1 (1,1) |  | 1 (1,1) |  | 1 (1,1) | 1 (1,1) | 1 (1,1) | |
| Rural | **0.17 (0.07, 0.44)** | ***p = 0.000****** | 0.55 (0.27, 1.11) | ***p =*** *0.097* | 0.26 (0.05, 1.27) | 7.62 (0.20, 295.12) | **0.39 (0.20, 0.75)***** | |
| **Contextual Regions** |  |  |  |  |  |  |  | |
| Agrarian (ref) | 1 (1,1) |  | 1 (1,1) |  | 1 (1,1) | 1 (1,1) | 1 (1,1) | |
| Pastoralist | 0.53 (0.26, 1.09) | *p = 0.084* | 1.28 (0.50, 3.24) | *p = 0.603* | 0.48 (0.22, 1.04) | 2.71 (0.34, 21.79) | 1.51 (0.54, 4.23) | |
| City dweller’s | 3.04 (0.60, 15.38) | *p = 0.178* | **3.65 (1.33, 10.06)** | ***p = 0.012***** | 2.89 (0.32, 25.95) | 1.10 (0.02, 71.70) | **2.96 (1.13, 7.77)**** | |
| Intraclass Correlation | 0.40 |  | 0.36 |  | 0.40 | 0.65 | 0.32 | |
| Akaike Information Criterion | 1604.09 |  | 1114.15 |  | *1064.39* | *607.78* | *1047.41* | |

NB. sig. at **sig. at 5% level; at ***sig. at 1% level; ref = reference group; n = Number of participants., HH = household wealth; AOR = Adjusted Odds ratios; Models adjusted for: Mother’s age, birth order, husband’s education, media exposure, decision making autonomy, place of residence, and contextual regions. Education adjusted in the models while stratified by wealth index and vice versa (-).

Additional file Table S4b. Multiple multilevel logistic regression exploring association between maternal exposure to sexual IPV and uptake of Health Facility Delivery stratified by education or income status for 2863 currently married women: 2016 Ethiopia DHS.

| Maternal health outcome | **Health facility Delivery** | | | | | | | |
| --- | --- | --- | --- | --- | --- | --- | --- | --- |
| Stratified by | **Education status** | | | | **HH wealth status** | | | |
| Stratum | No education (n=1788) | | *Primary or above (n=1075)* | | Low HH wealth (n=1496) | Medium HH wealth (n=433) | *High HH wealth index (n=934)* | |
| **Violence type** | AOR (95 % CI) | ***P-value*** | AOR (95 % CI) | ***P-value*** | AOR (95 % CI) |  | AOR (95 % CI) | |
| **Sexual IPV** |  |  |  |  |  |  |  | |
| No (ref) | 1 (1,1) |  | 1 (1,1) |  | 1 (1,1) | 1 (1,1) | 1 (1,1) | |
| Yes | 1.63 (0.92, 2.89) | *p = 0.097* | 1.25 (0.54, 2.92) | *p = 0.602* | 1.50 (0.75, 3.01) | 0.82 (0.27, 2.51) | **2.96 (1.02, 8.61)**** | |
| **Age** (years) |  |  |  |  |  |  |  |  |
| 15—24 (ref) | 1 (1,1) |  | 1 (1,1) |  | 1 (1,1) | 1 (1,1) | 1 (1,1) | |
| 25—34 | 0.99 (0.51, 1.92) | *p = 0.976* | 1.45 (0.80, 2.64) | *p = 0.226* | 1.20 (0.62, 2.34) | 0.68 (0.23, 1.98) | 1.77 (0.77, 4.06) | |
| 35—49 | 1.68 (0.78, 3.60) | *p = 0.184* | 1.40 (0.53, 3.72) | *p = 0.497* | 1.68 (0.70, 4.00) | 0.78 (0.19, 3.27) | 1.91 (0.68, 5.36) | |
| **Order of last birth** |  |  |  |  |  |  |  | |
| First (ref) | 1 (1,1) |  | 1 (1,1) |  | 1 (1,1) | 1 (1,1) | 1 (1,1) | |
| Second or third | 0.31 (0.15, 0.64) | ***p = 0.002****** | **0.51 (0.29, 0.91)** | ***p = 0.022***** | **0.40 (0.19, 0.82)**** | 0.50 (0.16, 1.56) | **0.18 (0.08, 0.43)***** | |
| Fourth or higher | 0.18 (0.08, 0.40) | ***p = 0.000****** | **0.13 (0.06, 0.28)** | ***p = 0.000****** | **0.10 (0.04, 0.23)***** | 0.59 (0.17, 2.07) | **0.05 (0.02, 0.14)***** | |
| **Education level of the women** |  |  |  |  |  |  |  | |
| No education (ref) |  |  |  |  | 1 (1,1) | 1 (1,1) | 1 (1,1) | |
| Primary and above education | - | ***-*** | - | *-* | 1.24 (0.71, 2.15) | **2.67 (1.22, 5.83)**** | 1.40 (0.77, 2.55) | |
| **Education level of their partners** | **-** | ***-*** | - | *-* |  |  |  | |
| No education (ref) | 1 (1,1) |  | 1 (1,1) |  | 1 (1,1) | 1 (1,1) | 1 (1,1) | |
| Primary and above | 1.16 (0.80, 1.69) | *p = 0.428* | 1.13 (0.61, 2.09) | *p = 0.697* | 0.85 (0.51, 1.42) | **2.06 (1.07, 3.97)**** | 1.53 (0.80, 2.90 | |
| **Exposure to mass media** |  |  |  |  |  |  |  | |
| No exposure (ref) | 1 (1,1) |  | 1 (1,1) |  | 1 (1,1) | 1 (1,1) | 1 (1,1) | |
| Exposed to either radio or TV (19.2%) | 1.07 (0.66, 1.76) | *p = 0.773* | 0.88 (0.50, 1.53) | *p = 0.642* | **3.07 (1.51, 6.25)***** | **0.42 (0.18, 1.00)**** | 0.83 (0.45, 1.55) | |
| Exposed to both radio and TV (13.5%) | **2.37 (1.25, 4.48)** | ***p = 0.008****** | **4.05 (1.93, 8.50)** | ***p = 0.000****** | 2.16 (0.71, 6.57) | 1.84 (0.52, 6.48) | **3.20 (1.63, 6.29)***** | |
| **Household wealth index** |  |  |  |  |  |  |  | |
| Low household wealth status (ref) | 1 (1,1) |  | 1 (1,1) |  | **-** | - |  | |
| Medium household Wealth status | 1.07 (0.71, 1.62) | *p = 0.739* | **2.85 (1.45, 5.60)** | ***p = 0.002****** | **-** | - |  | |
| High household Wealth status | 1.04 (0.64, 1.67) | *p = 0.887* | **3.12 (1.63, 5.97)** | ***p = 0.001****** | **-** | - | - | |
| **Decision-making Autonomy**^c^ |  |  |  |  |  |  |  | |
| No autonomy (ref) | 1 (1,1) |  | 1 (1,1) |  | 1 (1,1) | 1 (1,1) | 1 (1,1) | |
| Medium autonomy | **2.50 (1.31, 4.77)** | ***p = 0.005****** | 2.38 (0.87, 6.53) | *p = 0.092* | 1.11 (0.48, 2.55) | **3.47 (1.08, 11.15)**** | **6.72 (1.72, 26.26)***** | |
| High autonomy | **2.15 (1.20, 3.87)** | ***p = 0.011***** | 1.36 (0.53, 3.51) | *p = 0.526* | 1.03 (0.48, 2.20) | **2.84 (1.04, 7.75)**** | 3.15 (0.85, 11.63) | |
| **Place of residence** |  |  |  |  |  |  |  | |
| Urban (ref) | 1 (1,1) |  | 1 (1,1) |  | 1 (1,1) | 1 (1,1) | 1 (1,1) | |
| Rural | **0.04 (0.01, 0.13)** | ***p = 0.000****** | 0.05 (0.02, 0.14) | ***p = 0.000****** | **0.06 (0.01, 0.45)***** | 2.72 (0.08, 90.99) | **0.03 (0.01, 0.10)***** | |
| **Contextual Regions** |  |  |  |  |  |  |  | |
| Agrarian (ref) | 1 (1,1) |  | 1 (1,1) |  | 1 (1,1) | 1 (1,1) | 1 (1,1) | |
| Pastoralist | 0.25 (0.10, 0.62) | ***p = 0.003****** | 0.62 (0.19, 2.00) | *p = 0.425* | **0.28 (0.10, 0.74)**** | 1.20 (0.15, 9.71) | 0.51 (0.13, 2.07) | |
| City dweller’s | 0.73 (0.10, 5.51) | *p = 0.761* | 4.44 (0.55, 35.54) | *p = 0.160* | 0.79 (0.04, 15.11) | 0.64 (0.01, 50.27) | 2.72 (0.40, 18.38) | |
| Intraclass Correlation | 0.56 |  | 0.56 |  | 0.60 | 0.66 | 0.55 | |
| Akaike Information Criterion | 1446.44 |  | 918.75 |  | *938.04* | *624.96* | *768.06* | |

NB. sig. at **sig. at 5% level; at ***sig. at 1% level; ref = reference group; n = Number of participants., HH = household wealth; AOR = Adjusted Odds ratios; Models adjusted for: Mother’s age, birth order, husband’s education, media exposure, decision making autonomy, place of residence, and contextual regions. Education adjusted in the models while stratified by wealth index and vice versa (-).

Additional file Table S4c. Multiple multilevel logistic regression exploring association between maternal exposure to any forms of IPV and uptake of adequate ANC visits stratified by education or income status for 2863 currently married women: 2016 Ethiopia DHS.

| Maternal health outcome | **Adequate ANC Visits** | | | | | | | |
| --- | --- | --- | --- | --- | --- | --- | --- | --- |
| Stratified by | **Education status** | | | | **HH wealth status** | | | |
| Stratum | No education (n=1788) | | *Primary or above (n=1075)* | | Low HH wealth (n=1496) | Medium HH wealth (n=433) | *High HH wealth index (n=934)* | |
| **Violence type** | AOR (95 % CI) | ***P-value*** | AOR (95 % CI) | ***P-value*** | AOR (95 % CI) |  | AOR (95 % CI) | |
| **Any IPV** |  |  |  |  |  |  |  | |
| No (ref) | 1 (1,1) |  | 1 (1,1) |  | 1 (1,1) | 1 (1,1) | 1 (1,1) | |
| Yes | **0.50 (0.28, 0.87)** | ***p = 0.015***** | 1.25 (0.70, 2.23) | *p = 0.460* | 0.75 (0.40, 1.41) | 0.72 (0.23, 2.24) | 0.75 (0.40, 1.38) | |
| **Age** (years) |  |  |  |  |  |  |  |  |
| 15—24 (ref) | 1 (1,1) |  | 1 (1,1) |  | 1 (1,1) | 1 (1,1) | 1 (1,1) | |
| 25—34 | 1.23 (0.67, 2.26) | *p = 0.503* | **1.75 (1.07, 2.86)** | ***p = 0.025***** | **2.94 (1.59, 5.42)***** | 2.63 (0.88, 7.82) | 0.86 (0.48, 1.53) | |
| 35—49 | 1.25 (0.63, 2.51) | *p = 0.525* | **2.53 (1.16, 5.50)** | ***p = 0.020***** | **2.20 (1.03, 4.73)**** | **5.64 (1.29, 24.65)**** | 1.46 (0.68, 3.13) | |
| **Order of last birth** |  |  |  |  |  |  |  | |
| First (ref) | 1 (1,1) |  | 1 (1,1) |  | 1 (1,1) | 1 (1,1) | 1 (1,1) | |
| Second or third | 1.50 (0.72, 3.11) | ***p =*** *0.281* | 0.87 (0.55, 1.38) | *p = 0.563* | 1.09 (0.57, 2.10) | 1.24 (0.38, 4.04) | 0.78 (0.46, 1.34) | |
| Fourth or higher | 1.07 (0.49, 2.31) | *p = 0.870* | **0.54 (0.29, 0.99)** | ***p = 0.048***** | **0.43 (0.20, 0.91)**** | 1.04 (0.26, 3.97) | 0.63 (0.33, 1.20) | |
| **Education level of the women** |  |  |  |  |  |  |  | |
| No education (ref) |  |  |  |  | 1 (1,1) | 1 (1,1) | 1 (1,1) | |
| Primary and above education | - | ***-*** | - | *-* | 0.97 (0.58, 1.62) | **7.58 (3.18, 18.05)***** | **2.39 (1.50, 3.83)***** | |
| **Education level of their partners** | **-** | ***-*** | - | *-* |  |  |  | |
| No education (ref) | 1 (1,1) |  | 1 (1,1) |  | 1 (1,1) | 1 (1,1) | 1 (1,1) | |
| Primary and above | **1.60 (1.15, 2.23)** | ***p = 0.005****** | **2.23 (1.33, 3.75)** | ***p = 0.002****** | 0.79 (0.51, 1.22) | **3.64 (1.89, 7.00)***** | **2.26 (1.36, 3.77)***** | |
| **Exposure to mass media** |  |  |  |  |  |  |  | |
| No exposure (ref) | 1 (1,1) |  | 1 (1,1) |  | 1 (1,1) | 1 (1,1) | 1 (1,1) | |
| Exposed to either radio or TV (19.2%) | 1.20 (0.78, 1.86) | *p = 0.403* | 1.05 (0.65, 1.69) | *p = 0.837* | **1.87 (1.03, 3.39)**** | 1.26 (0.57, 2.76) | 0.82 (0.51, 1.33) | |
| Exposed to both radio and TV (13.5%) | **2.92 (1.61, 5.28)** | ***p = 0.001****** | 1.02 (0.59, 1.76) | *0.947* | 1.58 (0.67, 3.71) | 2.16 (0.63, 7.40) | 1.40 (0.85, 2.31) | |
| **Household wealth index** |  |  |  |  |  |  |  | |
| Low household wealth status (ref) | 1 (1,1) |  | 1 (1,1) |  | **-** | - |  | |
| Medium household Wealth status | 1.02 (0.70, 1.48) | ***p =*** *0.913* | **4.10 (2.25, 7.48)** | ***p = 0.000****** | **-** | - |  | |
| High household Wealth status | **0.54 (0.35, 0.85)** | ***p = 0.007****** | **2.85 (1.64, 4.94)** | ***p = 0.000****** | **-** | - | - | |
| **Decision-making Autonomy**^c^ |  |  |  |  |  |  |  | |
| No autonomy (ref) | 1 (1,1) |  | 1 (1,1) |  | 1 (1,1) | 1 (1,1) | 1 (1,1) | |
| Medium autonomy | **5.12 (2.72, 9.64)** | ***p = 0.000****** | 2.04 (0.83, 5.01) | *p = 0.120* | **2.92 (1.34, 6.34)***** | **5.74 (1.72, 19.21)***** | **4.27 (1.30, 14.06)**** | |
| High autonomy | **3.93 (2.18, 7.07)** | ***p = 0.000****** | **3.29 (1.41, 7.65)** | ***p = 0.006****** | **2.81 (1.37, 5.77)***** | **4.96 (1.70, 14.48)***** | **4.74 (1.50, 15.05)***** | |
| **Place of residence** |  |  |  |  |  |  |  | |
| Urban (ref) | 1 (1,1) |  | 1 (1,1) |  | 1 (1,1) | 1 (1,1) | 1 (1,1) | |
| Rural | **0.16 (0.06, 0.42)** | ***p = 0.000****** | 0.55 (0.28, 1.11) | ***p =*** *0.095* | 0.26 (0.05, 1.25) | 6.99 (0.18, 271.96) | **0.37 (0.19, 0.71)***** | |
| **Contextual Regions** |  |  |  |  |  |  |  | |
| Agrarian (ref) | 1 (1,1) |  | 1 (1,1) |  | 1 (1,1) | 1 (1,1) | 1 (1,1) | |
| Pastoralist | 0.55 (0.27, 1.13) | *p = 0.103* | 1.28 (0.51, 3.24) | *p = 0.599* | 0.48 (0.22, 1.04) | 2.83 (0.35, 22.92) | 1.50 (0.53, 4.20) | |
| City dweller’s | 3.10 (0.60, 15.89) | *p = 0.175* | **3.65 (1.33, 9.99)** | ***p = 0.012***** | 2.99 (0.33, 26.77) | 1.21 (0.02, 79.02) | **2.70 (1.03, 7.08)**** | |
| Intraclass Correlation | 0.40 |  | 0.36 |  | 0.40 | 0.65 | 0.32 | |
| Akaike Information Criterion | 1598.22 |  | 1113.63 |  | *1063.91* | *607.49* | *1050.76* | |

NB. sig. at **sig. at 5% level; at ***sig. at 1% level; ref = reference group; n = Number of participants., HH = household wealth; AOR = Adjusted Odds ratios; Models adjusted for: Mother’s age, birth order, husband’s education, media exposure, decision making autonomy, place of residence, and contextual regions. Education adjusted in the models while stratified by wealth index and vice versa (-).
