# Supplementary material for: Maternal exposure to intimate partner violence and uptake of maternal healthcare services in Ethiopia: Evidence from a national survey
Source: PLoS One. 2022 Aug 18;17(8):e0273146. doi: 10.1371/journal.pone.0273146 (PMC9387817; doi:10.1371/journal.pone.0273146)
Supplement: S4 Table — (DOCX) [file pone.0273146.s005.docx]

Additional file Table S5.

1. Effect of physical IPV on ANC when mother education is low compared to the effect when her education is higher and/or residing in poor household compared to medium and richest: 2016 Ethiopia DHS.
2. Marginal effect for education

B. Marginal effect for wealth status

1. Effect of sexual IPV on HFD when mother education is low compared to the effect when her education is higher and/or residing in poor household compared to medium and richest: 2016 Ethiopia DHS.
2. Marginal effect for education

B. Marginal effect for wealth status

1. Effect of any forms IPV on ANC when mother education is low compared to the effect when her education is higher and/or residing in poor household compared to medium and richest: 2016 Ethiopia DHS.
2. Marginal effect for education

B. Marginal effect for wealth status
